# Supplementary material for: Monitoring the bioenergetic state and cell lysis of Bacillus subtilis throughout the growth cycle
Source: Microb Cell Fact. 2026 Aug 12;25:232. doi: 10.1186/s12934-026-03086-8 (PMC13749677; doi:10.1186/s12934-026-03086-8)
Supplement: Supplementary file 1 [file 12934_2026_3086_MOESM2_ESM.docx]

**Monitoring the bioenergetic state and cell lysis of *Bacillus subtilis* throughout the growth cycle**

-supplementary material-

Maria Dakes Stavrakakis^1^, Taylor Wellfare Reid^1^, Madeleine Humphrey^1^, Tjeerd van Rij^2^, Colin R. Harwood^1^*, and Henrik Strahl^1^*

^1^ Centre for Bacterial Cell Biology, Biosciences Institute, Newcastle University, Richardson Road, Newcastle upon Tyne, NE2 4AX, United Kingdom

^2^ DSM Biotechnology Center, Alexander Fleminglaan 1, Delft 2613 AX, Netherlands

*to whom correspondence should be addressed:

Colin Harwood: colin.harwood@newcastle.ac.uk

Henrik Strahl: h.strahl@newcastle.ac.uk

**Figures**

**Supplementary Figure 1: *B. subtilis* strain MDS23 was generated by transforming strain 168 (*trpC2*) with a PCR-generated DNA from the *trpC* gene of NCIB3610T and selection for prototrophic transformants**

**(A)** Growth kinetics (OD_600_) of strains 168 and MDS23 in modified SMM + glucose (0.5% v/v). The growth of strain 168 with (green) and without (red) tryptophan (0.002% v/v) confirms its tryptophan auxotropy. The growth of MDS23 with or without tryptophan (black) is identical to that of 168 (green) with tryptophan, confirming the loss of tryptophan auxotrophy. The growth data is the average of three independent cultures. **(B)** The nucleotide sequence of the *trpC2* gene in strain 168 compared to the prototrophic (*trpC*) version of the gene in MDS23. The red box marks the missing TAA codon. **(c)** The derived amino acid sequences of the TrpC2 and TrpC proteins of strains 168 and MDS23, respectively. The sequence shows the position of the missing Ile residue (red box) at position 111, located at the junction of the β3 strand and α3 helix. Although not directly involved in forming the active site, the deletion abolishes the enzyme activity (52).

**Supplementary Figure 2: Continuous monitoring of lysis in a growing *B. subtilis* culture using SYTOX Green or Propidium Iodide is not feasible**

Growth kinetics (OD_600_) and fluorescence measurements of *B. subtilis* in mSMM with the presence of either **(A)** 1 μM SYTOX Green or **(B)** 10 μM propidium iodide. Graphs depict the means and standard deviation from three technical replicates. Strain used: *B. subtilis* prototroph (MDS23).

**Supplementary Figure 3: The deletion of two major *B. subtilis* nucleases does not significantly affect culture supernatant DNA levels**

**(A)** Growth kinetics (OD_600_) of wild type, Δ*nucA* and Δ*nucB* in mSMM at 37°C with the black arrow indicating the time point of sample collection. **(B)** SYTOX Green fluorescence signal of supernatants derived from an early stationary phase wild type, Δ*nucA* and Δ*nucB* cultures. The graph depicts the mean and standard deviation from three independent biological replicates, along with significance evaluated using a one-way, unpaired ANOVA. ns indicates a non-significant difference. **(c)** Agarose gel depicting DNA stained with Nancy-520 one of the supernatant samples shown in panel A. The 1 kb DNA Ladder from NEB® was used as a molecular size marker. Strains used: *B. subtilis* prototroph (MDS23), Δ*nucA* (MDS29) and Δ*nucB* (MDS30).

**Supplementary Figure 4: Fragmentation of DNA by sonication does not significantly affect the SYTOX Green fluorescence signals**

**(A)** SYTOX Green fluorescence signal of 5 technical replicates of *B. subtilis* wild type genomic DNA before and after sonication at 40% amplitude for 10 min. **(B)** The graph depicts the mean and standard deviation of 5 technical replicates, with significance assessed using a one-way, unpaired ANOVA. ns indicates a non-significant difference. **(c)** Agarose gel depicting DNA stained with Nancy-520 in the samples shown in panel **(A)**. The 1 kb DNA Ladder from NEB® was used as a molecular size marker. The contrast setting of the agarose gel image has been adjusted to enhance the visualisation of heavily fragmented DNA.

**Supplementary Figure 5: Culture supernatant DNA is not significantly degraded**

(A) Growth kinetics (OD_600_) of a multiple autolytic enzyme-deficient strain (Δ*lytABCDEF*) in mSMM at 37°C. The arrows indicate time points of culture supernatant sampling. (B) SYTOX Green fluorescence of culture supernatants with and without addition of exogenous DNA (*B. subtilis* MDS23 chromosomal DNA, 33 ng/ul) and 16 h incubation at 37⁰C. The graph depicts the mean and standard deviation of three technical replicates. (C) Agarose gel showing DNA stained with Nancy-520 of the supernatant samples shown in panel B. The 1 kb DNA Ladder from NEB® was used as a molecular size marker. Note the lack of visible degradation. (D) Autofluorescence levels for the samples shown in panel B, measured in the absence of SYTOX Green. Note the negligible autofluorescence levels measured in the absence of SYTOX Green. Strains used: *B. subtilis* prototroph (MDS23) as a source of exogenous DNA and KS19 (Δ*lytABCDEF*).

**Movie S1: Autolysis of *B. subtilis* can be monitored with the membrane permeability-indicator SYTOX Green**

*B. subtilis* phase contrast and fluorescence time-lapse microscopy of cells incubated on LB time-lapse slides and stained with 200 nM SYTOX Green in the presence of 100 μM of the proton uncoupler CCCP. Strain used: *B. subtilis* wild type (168).
